# 

**Published:** 1899-10

**Authors:** 


TABLE OF CONTENTS ON LAST ADVERTISING PAGE.

Vol. XV. OCTOBER, 1899. No. 4.

LIBRARY
[illegible]
3. 10

### TEXAS
### Medical Journal.

Subscription, $1.00 a Year, in Advance.

Single Copies, 10 Cents.

PUBLISHED AT AUSTIN, TEXAS, BY
F. E. DANIEL, M. D., & S. E. HUDSON, M. D.,
Proprietors.

Eastern Representative: John Guy Monihan, St. Paul Building, 220 Broadway, New York City.

Entered at the Postoffice at Austin, Texas, as Second-class Mail Matter.

